# Prospective Cohort Study on Refractive Changes after Trabeculectomy

**DOI:** 10.1155/2019/4731653

**Published:** 2019-08-07

**Authors:** Kentaro Iwasaki, Yoshihiro Takamura, Shogo Arimura, Takahiro Tsuji, Takehiro Matsumura, Makoto Gozawa, Masaru Inatani

**Affiliations:** Department of Ophthalmology, Faculty of Medical Sciences, University of Fukui, Fukui 910-1193, Japan

## Abstract

We prospectively evaluated refractive changes in the eyes of 97 patients who underwent trabeculectomy at Fukui University Hospital, Fukui, Japan. The primary outcome measure was the refractive change after trabeculectomy. Secondary outcome measures included postoperative complications and prognostic factors for refractive change. We observed a progressive and significant mean refractive myopic shift of −0.80 D at 12 months after surgery. In phakic eyes, the mean myopic refractive shifts progressed significantly by −0.46 D at 3 months after surgery (*P*=0.003), by −0.52 D at 6 months (*P*=0.012), and by −1.31 D at 12 months (*P* < 0.001). In the pseudophakic eyes, we found no significant refraction progression at any of the postsurgery follow-up visits. Our multivariable analyses showed that lens nuclear color grade change was a significant prognostic factor for refractive myopic progression (*P* < 0.001). Trabeculectomy causes refractive myopic progression in phakic eyes. Nuclear sclerotic cataract progression is associated with refractive myopic shift after trabeculectomy. This trail is registered with UMIN000007813.

## 1. Introduction

Trabeculectomy is a common filtering surgery for patients with glaucoma and medically uncontrollable intraocular pressure (IOP). Patients treated with trabeculectomy often encounter postoperative visual disturbances. Hypotonic maculopathy, postoperative hyphema, anterior chamber inflammation, and fixation loss or wipeout diminish visual acuity after trabeculectomy [1].

In terms of refraction, trabeculectomy causes corneal astigmatism, which also deteriorates visual acuity [2–7]. Our study compared trabeculectomy to Ex-PRESS filtering surgery and showed that postoperative nuclear cataract progression occurs more frequently after trabeculectomy than after Ex-PRESS filtering surgery [8]. Moreover, a collaborative normal tension glaucoma study has shown that IOP-lowering treatment protects visual field loss in eyes with normal tension glaucoma. However, cataract progression causes visual disturbances in eyes treated with trabeculectomy [9]. Nuclear cataract not only causes visual disturbances but also induces myopic changes in senior patients [10]. In addition to trabeculectomy, eyes treated with vitrectomy exhibit myopic changes due to cataract progression, resulting in deteriorated visual acuity [11–14]. Despite cataract progression being common after trabeculectomy, refractive changes have not been prospectively quantified in treated eyes. Therefore, we designed this study to evaluate the refractive changes after trabeculectomy and to identify the patient-related factors for the refractive change.

## 2. Materials and Methods

### 2.1. Patient Selection

This was a prospective clinical cohort study that was approved by the institutional review board of the Fukui University Hospital (Fukui, Japan). We registered this study with the University Hospital Medical Information Network Clinical Trials Registry of Japan UMIN000007813; date of access and registration: April 24, 2012). Our study protocol adhered to the tenets of the Declaration of Helsinki. We obtained written informed consent from all patients.

This prospective study evaluated refractive changes after trabeculectomy. We recruited patients between April 2012 and March 2016 at the Fukui University Hospital. The inclusion criteria were being aged ≥20 years and having open angle glaucoma (primary open angle, exfoliation, or uveitic glaucoma) without a history of intraocular surgery other than phacoemulsification. The exclusion criteria were patients with aphakic eyes, eyes with a history of glaucoma surgery before trabeculectomy, eyes with previous vitrectomy, or pseudophakic eyes previously treated with cataract extraction other than phacoemulsification.

### 2.2. Surgical Procedures

All surgeries were performed by one surgeon (MI). The surgeon made either a 5 mm conjunctival incision along the limbus to create a fornix-based conjunctival flap, or an 8 mm conjunctival incision parallel to the limbus at 7–9 mm posterior to the limbus to create a limbus-based conjunctival flap. A 4 mm wide half layer scleral flap was also created. Mitomycin C (0.4 mg/ml) was applied on and under the scleral flap and under Tenon's capsule for 4 min, and the eye was irrigated with physiological saline (100 ml). The surgeon then excised a deep limbal block to create a fistula in the anterior chamber to proceed with peripheral iridectomy. The surgeon closed the scleral flap by using three sutures of 10-0 monofilament nylon. The conjunctival flap was also sutured with 10-0 monofilament nylon. All patients received similar postoperative topical medications with 0.5% levofloxacin 3 times a day for 1 month and 0.1% betamethasone sodium phosphate 3 times a day for 6 months.

### 2.3. Data Collection

We collected patient data including gender, age, glaucoma type, preoperative IOP, postoperative IOP, refractive errors, best corrected visual acuity (BCVA), number of glaucoma medications, anterior chamber depth, axial length, anterior chamber opening duration, and presence of postoperative complications. We used the logarithm of the reciprocal of the decimal BCVA to approximate the logarithm of the minimal angle of resolution (LogMAR). We scheduled the first study-related visit 2 weeks after surgery; thereafter, follow-up visits occurred 1 month, 3 months, 6 months, and 12 months after surgery. We assessed IOP, BCVA, refractive errors, and number of glaucoma medications before surgery and at all follow-up visits. We also looked for complications at all follow-up visits. We measured preoperative and postoperative refractive errors in all eyes by using an automatic refractometer (TONOREFII; NIDEK, Aichi, Japan). Refractive errors were measured three times at each visit, and the average of three refractive errors was used as the refraction value for analysis. We defined the anterior chamber open-time during the surgery as the time from the initial fistula creation incision in the anterior chamber to the scleral flap suturing. We quantified the nuclear cataract progression by using the Lens Opacification Classification System III (LOCS-III) nuclear color grade [15]. The definition of postoperative complications was as follows: shallow anterior chamber was identified if the anterior chamber at the pupillary border of the iris was narrower than the corneal thickness or if cornea-iris contact was observed at the peripheral anterior chamber and hypotony was defined as IOP <5 mmHg on two consecutive follow-up visits after 3 months.

### 2.4. Outcome Measures

The primary outcome measure was the refractive change after trabeculectomy. Secondary outcome measures included postoperative complications and prognostic factors for refractive changes.

### 2.5. Statistical Analysis

We performed univariate comparisons between groups using paired *t* test with Bonferroni correction, unpaired *t*, and chi-squared tests. The longitudinal repeated measures were analyzed using one-way repeated measures analysis of variance (ANOVA). We performed multivariate analysis to determine the prognostic factors for myopic progression using a logistic regression model. We considered *P* values <0.05 as statistically significant.

## 3. Results

### 3.1. Patient Characteristics

We enrolled a total of 106 patients (106 eyes) in this study. Five patients were not available for analysis within the 1-year follow-up. We excluded four patients from the analysis because of glaucoma reoperations within the 1-year follow-up. In total, we evaluated 97 patients (97 eyes) for the study.  Table 1 summarizes the patients' characteristics.

### 3.2. Primary Outcome Measure

Table 2 demonstrates the time course for the refractive changes occurring after trabeculectomy. The mean refraction in all the eyes was −1.93 ± 3.78 D before surgery, and a refractive myopic shift progressed significantly to −2.22 ± 3.72 D at 1 month after surgery (−0.29 progression; *P*=0.016), to −2.22 ± 3.89 D at 3 months after surgery (−0.29 progression; *P*=0.02), to −2.28 ± 3.82 D at 6 months after surgery (−0.35 progression; *P*=0.016), and to −2.73 ± 3.90 D at 12 months after surgery (−0.80 progression; *P* < 0.001).

### 3.3. Subgroup Analyses of the Primary Outcome

We divided patients into phakic and pseudophakic groups and conducted subgroup analyses. In the phakic group, the refraction was −2.31 ± 4.49 D before surgery and the refractive myopic shift progressed significantly to −2.77 ± 4.56 D at 3 months after surgery (−0.46 progression; *P*=0.008), to −2.83 ± 4.50 D at 6 months after surgery (−0.52 progression; *P*=0.012), and to −3.62 ± 4.51 D at 12 months after surgery (−1.31 progression; *P* < 0.001). In the pseudophakic group, we found no significant progression of refraction at any of the postsurgery follow-up visits (Table 2).

### 3.4. Secondary Outcome Measures

#### 3.4.1. Determinants of Refractive Myopic Progression in Phakic Eyes

We divided phakic patients (*n* = 61) into those with myopic progression (*n* = 22) and those without it (*n* = 39) and conducted subgroup analyses. We defined myopic progression as >−1 D progression at 12 months after surgery from the preoperative refraction value. We compared various factors between the two groups (Table 3). The patients with myopic progression were significantly older than those without it (*P*=0.003). The preoperative and postoperative BCVAs in the patients with myopic progression were significantly worse than those in patients without it (Pre, *P*=0.03; Post, *P* < 0.001). The exfoliation glaucoma and fornix-based incisions in the patients with myopic progression were significantly more frequent than those in the patients without myopic progression (*P* < 0.001 and *P*=0.017, respectively). The axial length in the patients with myopic progression was significantly shorter than that in the patients without it (*P*=0.018). The preoperative and postoperative nuclear color grades in the patients with myopic progression were significantly higher than those in the patients without it (Pre; *P*=0.004, Post; *P* < 0.001). Finally, the nuclear color grade changes (the difference between the preoperative and the postoperative nuclear color grades) in the patients with myopic progression were significantly higher than those in the patients without myopic progression (*P* < 0.001).

Table 4 shows the postoperative complications in phakic eyes. In terms of postoperative complications, we found no significant differences between the patients with myopic progression and those without it.

We evaluated patient characteristics, including age, type of glaucoma, preoperative LogMAR BCVA, conjunctival incision, axial length, preoperative nuclear color grade, and change of nuclear color grade as possible determinants of refractive myopic progression. Our multivariate analyses using logistic regression models showed that a higher change of nuclear color grade was significantly associated with refractive myopic progression (*P* < 0.001; Table 5).

## 4. Discussion

The aims of our study were to evaluate refractive changes after trabeculectomy and to identify prognostic factors for postoperative myopia. A myopic shift in refraction progressed significantly by −0.80 D 12 months after trabeculectomy. In the phakic eyes, the myopic shift had progressed significantly by −0.46 D at 3 months after surgery (*P*=0.003), by −0.52 D at 6 months (*P*=0.012), and by −1.31 D at 12 months (*P* < 0.001), whereas there were no significant refraction changes in the pseudophakic eyes at any postsurgery follow-up visits. Our multivariable analyses confirmed that the nuclear sclerotic cataract progression was significantly associated with refractive myopic progression (*P* < 0.001).

As for refractive changes after trabeculectomy, corneal astigmatism has been evaluated by several studies [2–7]. Although cataract progression is a common late complication in eyes treated with trabeculectomy [1, 16–22], refractive changes due to cataract progression have not been prospectively analyzed. Our prospective study including phakic and pseudophakic eyes is unique because it clearly showed that nuclear sclerotic cataract progression after trabeculectomy causes myopia in eyes with glaucoma.

The reason for cataract progression after trabeculectomy remains unknown. Eyes with trabeculectomy have a higher risk of cataract progression compared with those with nonpenetrating deep sclerectomy [23]. A comparison of trabeculectomy and viscocanalostomy showed a higher tendency for cataract progression in eyes treated with trabeculectomy [24]. Hypotony in eyes with trabeculectomy may be related to cataract progression, and the lens-to-cornea contact due to anterior chamber shallowing causes cataract progression. Although we found no significant differences in the anterior chamber depths between the patients with and without myopic progression, the frequency of patients with shallow anterior chambers was 18.2% in those with myopic progression and only 7.7% in those without it. This suggests the shallow anterior chambers in patients with myopic progression may have contributed to cataract progression, resulting in a myopic shift in phakic eyes.

Other possible mechanisms for cataract progression after trabeculectomy include a reduction in aqueous humor production (which may reduce nutrient delivery to the lens), the toxicity of mitomycin C for lens epithelium [17], and the aqueous humor flow change due to peripheral iridectomy [25]. Compared with trabeculectomy, glaucoma surgeries without peripheral iridectomy such as nonpenetrating deep sclerectomy [23] and viscocanalostomy [24] are not associated with cataract progression. When prophylactic laser peripheral iridotomy was performed in eyes suspected of having primary angle closure, the eyes encountered a greater progression of cataract than the eyes without prophylactic laser peripheral iridotomy [25]. Ex-PRESS filtering surgery, a mitomycin C-augmented filtering surgery without peripheral iridectomy, offers less progression of nuclear cataract than trabeculectomy, while maintaining similar postoperative IOPs [8]. Taken together, the aqueous humor flow changes through the iris window after trabeculectomy may facilitate cataract progression in phakic eyes.

Exfoliation glaucoma and fornix-based incision have been associated with cataract progression after trabeculectomy [26–28]. Consistent with these studies, the number of eyes having myopic progression was significantly higher with exfoliation glaucoma and fornix-based incisions than eyes without myopic progression in this study. However, our multivariate analysis did not confirm these factors as prognostic for myopic progression. These results may be due to the associations of exfoliation glaucoma and fornix-based trabeculectomy with cataract progression that are not directly related to myopic progression.

Myopic progression occurs in eyes after lens-sparing vitrectomy due to nuclear sclerotic cataract progression [11–14], a result consistent with ours. However, the mechanism of cataract progression in eyes with vitrectomy is probably different from that of trabeculectomy. Cataract progression after lens-sparing vitrectomy has two possible mechanisms: intraoperative lens protein oxidation and surgery-induced alternation of the lens's biochemical microenvironment. Oxygen pressure becomes high after vitrectomy, which may cause oxidative damage to the crystalline lens [29]. Vitrectomy exposes the posterior part of the lens to increased oxygen, resulting in the formation of nuclear sclerotic cataracts [30, 31]. Oxidative stress at the posterior part of the lens does not seem to be related to cataract progression after trabeculectomy. The biochemical effects such as the perfusion solution [32], the aqueous humor dynamics [33], and a change of aqueous humor constitution [34, 35] are known to promote changes in lens metabolism after vitrectomy. By contrast, changes in the lens biochemical microenvironment after trabeculectomy remain unknown, but the aqueous humor flow change after trabeculectomy may cause such changes.

We are aware of the limitations of our study. First, we used LOCS-III to evaluate cataract grade and, thus, were unable to objectively evaluate the nuclear cataract grade. An anterior eye segment analysis system to evaluate the lens light scattering would have provided a more objective approach. Second, refraction after trabeculectomy is influenced by many factors, such as changes in the corneal topography and axial length. In the current study, we could not evaluate the astigmatism and axial length. The correlation between myopic shift and the change of astigmatism and axial length should be evaluated in future studies. Third, we measured all refractions without mydriasis. Although, the effect of accommodation was small because of the advanced ages of our patients, future studies with mydriatic refractions should be performed. Fourth, our data still do not offer long-term results for postoperative changes after 3 years or longer. The present study will be monitored for 5 years after surgery. Further follow-up periods might provide more information about refractive changes after trabeculectomy.

## 5. Conclusions

In all, trabeculectomy causes refractive myopic progression postoperatively in phakic eyes. Nuclear sclerotic cataract progression after trabeculectomy is the cause of refractive myopic progression.

## Figures and Tables

**Table 1:** Patient characteristics.

Characteristics	Total (*n* = 97)
Age, mean (SD) (years)	69.2 (13.1)
Gender, *n* (%)	
Men	46 (47)
Women	51 (53)
Type of glaucoma, *n* (%)	
Primary open angle glaucoma	49 (51)
Exfoliation glaucoma	42 (43)
Uveitic glaucoma	6 (6)
Preoperative IOP, mean (SD) (mmHg)	26.7 (9.6)
Number of preoperative glaucoma medications, mean (SD)	3.2 (1.0)
Postoperative IOP, mean (SD) (mmHg)	13.4 (3.7)
Number of postoperative glaucoma medications, mean (SD)	0.6 (1.3)
Conjunctival incision, *n* (%)	
Fornix-based	61 (63)
Limbus-based	36 (37)
Lens status, *n* (%)	
Phakic	61 (63)
Pseudophakic	36 (37)
Anterior chamber opening duration, mean (SD) (min)	5.0 (1.6)
Axial length, mean (SD) (mm)	24.4 (2.2)

IOP, intraocular pressure; SD, standard deviation.

**Table 2:** Time course of refractive changes after trabeculectomy.

(Diopters)	All (*n* = 97)	Phakic (*n* = 61)	Pseudophakic (*n* = 36)
Preoperative	−1.93 ± 3.78	−2.31 ± 4.49	−1.28 ± 1.95
1 month	−2.22 ± 3.72	−2.63 ± 4.41	−1.15 ± 1.91
3 months	−2.22 ± 3.89	−2.77 ± 4.56	−1.28 ± 2.03
6 months	−2.28 ± 3.82	−2.83 ± 4.50	−1.36 ± 2.01
12 months	−2.73 ± 3.90	−3.62 ± 4.51	−1.22 ± 1.78
*P* value			
All	0.70^a^	0.59^a^	0.97^a^
Pre vs. 1 month	0.016^b^	0.08^b^	0.33^b^
Pre vs. 3 months	0.02^b^	0.008^b^	>0.99^b^
Pre vs. 6 months	0.016^b^	0.012^b^	>0.99^b^
Pre vs. 12 months	<0.001^b^	<0.001^b^	>0.99^b^

Data expressed as mean ± standard deviation. *P* values: ^a^one-way repeated measures ANOVA; ^b^paired *t* test with Bonferroni correction.

**Table 3:** Comparison of patient characteristics between the patients with myopic progression and the patients without myopic progression.

Characteristics	Myopic progression (*n* = 22)	Without myopic progression (*n* = 39)	*P* value
Age, mean (SD) (years)	73.5 (8.6)	61.7 (14.6)	0.003
Gender, *n* (%)			0.18
Men	14 (64)	17 (44)	
Women	8 (36)	22 (56)	
Preoperative LogMAR BCVA, mean (SD)	0.3 (0.3)	0.2 (0.3)	0.03
Postoperative LogMAR BCVA, mean (SD)	0.9 (0.6)	0.3 (0.3)	<0.001
Glaucoma type, *n* (%)			<0.001
Exfoliation glaucoma	15 (68)	8 (20)	
Other glaucoma types	7 (32)	31 (80)	
Preoperative IOP, mean (SD) (mmHg)	27.3 (9.1)	24.8 (9.5)	0.16
Postoperative IOP, mean (SD) (mmHg)	14.0 (4.4)	13.2 (2.9)	0.50
Conjunctival incision, *n* (%)			0.017
Fornix-based	19 (86)	22 (56)	
Limbus-based	3 (14)	17 (44)	
Anterior chamber opening duration, mean (SD) (min)	5.2 (1.6)	5.3 (1.9)	0.86
Axial length, mean (SD) (mm)	23.5 (1.6)	24.8 (2.1)	0.018
Preoperative nuclear color grade	2.4 (0.7)	1.8 (0.8)	0.004
Postoperative nuclear color grade	3.6 (0.8)	2.0 (1.0)	<0.001
Nuclear color grade change	1.2 (0.8)	0.2 (0.5)	<0.001

BCVA, best corrected visual acuity; IOP, intraocular pressure; LogMAR, logarithm of minimum angle of resolution; SD, standard deviation.

**Table 4:** Postoperative complications in phakic eyes.

*n* (%)	Myopic progression (*n* = 22)	Without myopic progression (*n* = 39)	*P* value
Hyphema	6 (27)	8 (21)	0.55
Shallow anterior chamber	4 (18)	3 (8)	0.24
Choroidal detachment	4 (18)	4 (10)	0.44
Bleb infection	0 (0)	2 (5)	0.53
Hypotony	0 (0)	0 (0)	NA

NA, not applicable.

**Table 5:** Multivariate analysis for determining prognostic factors for refractive myopic progression after trabeculectomy using a logistic regression model.

Variable	RR (95% Cl)	*P* value
Type of glaucoma (exfoliation glaucoma/other)	1.94 (0.30–12.1)	0.47
Age per year	0.96 (0.86–1.07)	0.49
Preoperative LogMAR BCVA per 1.0	5.02 (0.41–81.7)	0.20
Conjunctival incision (fornix-based/limbus-based)	0.81 (0.09–6.26)	0.84
Axial length per mm	0.79 (0.42–1.38)	0.42
Preoperative nuclear color grade per 1.0	2.46 (0.73–9.63)	0.15
Nuclear color grade change per 1.0	12.1 (3.17–71.8)	<0.001

BCVA, best corrected visual acuity; 95% CI, 95% confidence interval; LogMAR, logarithm of minimum angle of resolution; RR, relative risk.

## Data Availability

All data generated or analyzed during this study are included in this published article.
